# The European Telecommunication Standards Institute (ETSI): in search for legitimacy and resilience of European standardisation

**DOI:** 10.1080/13511610.2024.2395262

**Published:** 2024-10-10

**Authors:** Olia Kanevskaia, Panagiotis Delimatsis, Stephanie Bijlmakers

**Affiliations:** a Department of International European Law and Utrecht Centre for Regulation and Enforcement in Europe (RENFORCE), Utrecht University, Utrecht, the Netherlands; b Tilburg Institute for Law, Technology and Society (TILT) and Tilburg Law and Economics Center (TILEC), Tilburg University, Tilburg, the Netherlands

**Keywords:** European standardisation strategy, harmonised standards, ETSI, telecommunications, legitimacy, organisational resilience

## Abstract

European standardisation, a key institution for the functioning of the EU single market, functions at the crossroads of private rule-making and public policy. Recently, it has been subjected to fierce scrutiny by the European Commission and the European Court of Justice. Expressed concerns relate to the legal value of standards and the legitimacy of institutions creating them but have broader implications for European competitiveness, highlighting the strategic role of standards. By far much of this critique has been directed towards the European Telecommunications Standards Institute (ETSI), the EU’s crown jewel of technology standardisation. ETSI’s normative power manifests itself in policymaking, legislation and market processes, rendering it an interesting organisation to study as a proxy to understand the legitimacy and resilience of European standardisation. Against this backdrop, this introduction sets the theoretical and legal context for this Special Issue. Upon discussing the background and dynamic of European standardisation, it provides a theoretical framework for ‘crises' and ‘legitimacy’, tailoring it to the particular case of ETSI and the challenges it faces at the policy and judicial level. Lastly, it provides a critical reflection on the Research Articles in this Special Issue, linking them to the current discourses on legitimacy and resilience.

## 1. Introduction

For a long time, technical standards have spurred discussions on innovation and economic advancement. Being ‘formalised sets of rules with a high degree of specificity’ (Djelic and Hond 2014), technical standards exist in virtually every sector. Standards enable long-term economic growth and knowledge-spillovers (Blind and Jungmittag 2008; Blind and Mangelsdorf 2016; Blind and von Laer 2022) and are crucial for the adoption and diffusion of new technologies (Swann 2000). Their functions range from purely coordinative to regulative, establishing the rules to follow for market actors (Delimatsis 2018). Being a product of science and expertise, standards are set in Standards Development Organizations (SDOs): institutions operating independently from the State machinery under private law and consisting predominantly of commercial actors.

Despite their ostensibly private nature, technical standards have managed to transform economies, institutions and society. Especially in the European Union (EU), standards have been a part and parcel of market integration and industrial policies (Egan 2001), making the three European SDOs (the European Committee for Standardization-CEN; the European Committee for Electrotechnical Standardization-CENELEC; and ETSI) increasingly relevant for the regulation and smooth operation of economic activity not only in Europe, but also globally. The history of European innovation is inseparable from European standardisation: take the example of the GSM standard developed by ETSI (Pelkmans 2001). After dominating global markets in late '80s, it has successfully evolved into 3G, 4G and currently 5G specifications, enabling wireless connections through cellular networks and linking millions of businesses and people. Standards such as 5G thus play not only strategic, but also societal roles, being crucial to our everyday activities.

In a world that strives for technological progress, standards also bring about political and societal challenges. As standards can both unite and divide (Wirth 2009), tensions arise among companies competing to drive the development of technology standards (Baron and Kanevskaia 2021), rendering standardisation an arena of power competition (Rühlig 2022). Being responsible for the development of global technologies, standard setters are endowed with power and authority (Mattli and Büthe 2003) and therefore challenge the current normative order. Furthermore, as standards are interwoven in the fabric of society, they also carry – and affect – our fundamental values, raising questions of legitimacy (Senden 2020). Similar questions of legitimacy linger as standardisation increasingly becomes instrumentalised by governments in their pursuit of digital autonomy, sovereignty or supremacy (i.e. Broeders, Cristiano, and Kaminska 2023; EC 2021), either when standards are incorporated in national regulations or when governments promote, be it directly or indirectly, specific standards in regional or international SDOs. SDOs are coordination devices where nonetheless strategic behaviour and opportunistic conduct may lead to results that are not always free from undue influence, leading to technically suboptimal and politically questionable outcomes (Delimatsis, Kanevskaia, and Verghese 2021). The current geopolitical rivalry, ranging from competition for access to semiconductor technology to Artificial Intelligence (AI), 5G or quantum computing standards, exacerbates the repercussions that SDO choices may have on global economic relations and often calls them to take sides in ring-fencing attempts made by States.

This Introduction proceeds as follows. First, we offer a succinct discussion relating to the evolution and functioning of the European standardisation system and how ESOs have interacted over time in order to understand the ecosystem within which ETSI has developed and grown. Second, we detail certain tentative conceptual premises of the Special Issue relating to legitimacy and legitimation, as well as authority and propose a dynamic view of such terms that may be challenged in certain time periods. Third, we focus more specifically on the 2022 Standardisation Strategy as a key inflection point for ESOs and ETSI in particular and attempt to distil the key objections that put ETSI in the spotlight. We conclude by providing a critical reflection on the contributions included in this Special Issue and how they advance the discussion in academic and policy circles about the legitimacy and resilience of ESOs, again focusing on ETSI as a prominent player in this ecosystem.

## 2. European standards bodies as emerging actors of global innovation

### 2.1. European standardisation in the historical context

The European standardisation model is based on a public-private partnership, where private ESOs are granted public authority to develop standards in support of European legislation. This model has proven successful, in a way that it allows a certain margin for the industry to decide which technical rules to abide by while ensuring that these rules are harmonised across the EU Member States and comply with non-negotiable health and safety requirements (Egan 2001; Schepel 2013). It has, however, also been subjected to criticism, especially with regard to the lack of inclusiveness and democratic oversight of ESOs standardisation processes (Gerardy 2023; Vaele and Borgesius 2021).

The European standardisation technique – the ‘New Approach’^1^ – was introduced in the 1980s as a response to the slow process of issuing technical specifications by the European Commission (Pelkmans 1987). It was later updated to the ‘New Legislative Framework’ (NLF)^2^ adjusting the requirements on market surveillance and conformity assessment. The New Approach/NLF formula goes as follows: the European Commission sets the essential requirements for health and safety in its Directives and Regulations, and the three ESOs produce harmonised standards, on the basis of a standardisation request by the Commission, detailing the technical aspects allowing to fulfil these essential requirements. Compliance with harmonised standards grants a presumption of conformity with the essential requirements of the Directive, a presumption that has legal effects although harmonised standards remain voluntary. ESOs thus have a significant role to play when it comes to the rulebook on market access and harmonisation within the EU.

This enrollment of private actors in the EU regulatory process has been a manifestation of so-called Europeanisation, a term that not only captures how EU law influences national practice but also how to address the lack of accountability and legitimacy of European institutions (Buller and Gamble 2002). Other than contributing significant technical expertise to the EU regulatory making in line with the tenets of neo-functionalism, the involvement of ESOs in the EU legislative process also allows for more participatory forms of governance and rule-making (Weatherill 2016). From a private governance perspective, though, such constellations give an important entry point for non-public organisations to influence regulatory outcomes (Delimatsis and Verghese 2024). Indeed, just like any private organisation that is involved in rulemaking, ESOs have a certain level of political agency and expressed policy preferences that they would like to see reflected in the policymaking process (Renckens 2020). The EU regulatory system in fact allows this type of interaction. Given the EU’s ambition to become a global actor in technological progress and to promote its standards at the global level (EC 2022b), it is not surprising that ESOs, and especially ETSI, are being regarded as enablers of European competitiveness in global technological rivalry.

### 2.2. Interaction among the ESOs

As the developers of harmonised European standards, ESOs have common policy objectives, including responding to the market needs and building consensus among standardisation stakeholders.^3^ While defined almost two decades before the European Standardisation Strategy came into being, these objectives already then referred to the EU’s aspirations to advance European competitiveness and promote the EU interests globally. In this regard, the institutional rules of ESOs should ensure that the organisations remain fully accountable to all interested EU parties, noting that they also carry responsibility to include broader interests, including those of workers, consumers or the environment.^4^

ESOs are also required to implement the widely accepted principles for standards development, including openness, transparency and coherence (WTO 2000). The latter is concretised in the 2003 Cooperation Guidelines:

Coherence, at all levels and between the three European Standards Organisations, should be ensured in the planning, execution and implementation of standardisation programmes and activities carried out by the European Standards Organisations, where applicable through the approval and implementation activities at national level.^5^

However, when it comes to cooperation between the ESOs (but also between the ESOs, the European Commission and the European Free Trade Association-EFTA), no general requirement for cooperation is present in the legislative framework. Neither is there a minimum threshold on what cooperation between the ESOs should entail. Rather, the national standardisation bodies and ESOs themselves are left to decide on the type of intra-ESO cooperation they desire, and how it should be achieved. The EU Standardisation Regulation is also silent on this issue, although it encourages ESOs to cooperate with international standard-setting organisations and even private consortia in the field of ICT to ensure coherence and avoid fragmentation or duplication.

The work of ESOs is divided across different sectors. CEN is predominantly active in such sectors as chemicals, food and construction;^6^ CENELEC’s work is more focused on, among others, electrical equipment, mechanical engineering and metals.^7^ At the same time, there is also a considerable overlap between the work of CEN and CENELEC, for instance in energy, transport and healthcare. ETSI, in turn, has traditionally been focused on wireless systems, ICT and interoperability standards.

Despite these jurisdictional differences, the work of the three ESOs is becoming increasingly overlapping, not least due to growing digitalisation. While in some sectors, the ESOs cooperate on joint projects (e.g. the Smart Meters Coordination Group),^8^ or collectively sign memoranda and cooperation agreements,^9^ in other areas, such as cybersecurity, standardisation work is conducted in parallel (Kamara 2024). At the same time, nothing in the respective operational framework of the three ESOs precludes them from becoming organisational partners or participating in each other’s work through technical bodies or advisory committees (for the account of initial coordination and collaboration among ESOs, see Egan 2024).

Amid the three ESOs, ETSI clearly stands out for its focus on technologies as well as its governance and decision-making. Unlike CEN and CENELEC, which are composed of standardisation committees of EU and European Economic Area (EEA) Member countries, ETSI’s membership also includes private companies (including from third countries) which can join with different national branches. According to the European Commission (EC 2022b), this governance model invites foreign interests to potentially dominate ETSI’s standard-setting processes, which then questions the extent to which European values are still preserved in European standardisation. In this setting, the recent election of a US-company affiliate as Chair of ETSI Board, ETSI’s executive organ, which, among others, approves the terms of reference for ETSI technical committees (TCs) and endorses the appointment of TC chairs, raised eyebrows.

The fact that commercial interests prevail in ESOs is not new; pre-eminence of foreign commercial interests, however, is yet a different stroke that deserves further attention. To be precise, the Commission doubts whether ETSI’s governance processes are sufficient to mitigate concerns arising from the prevalence of foreign commercial interests. In a broader sense, this calls into question ETSI’s commitment to promote European values and produce standards that support European policies.

## 3. Legitimacy, authority and crisis in European standardisation

Legitimacy is a multi-faceted and multi-dimensional analytical concept. In the case of private governance, any attempt to assess its quality typically entails a discussion about the sources of legitimacy of such modes of governance. Legitimacy in that sense is a dynamic concept and an empirically observable phenomenon. It has to be assessed and observed at any given moment in time and space, which in turn may mean that in certain points in time, higher or lower levels of legitimacy exist. An institution can gain, sustain, increase or lose its legitimacy (Tallberg and Zürn 2019).

Conceptually, legitimacy has a normative side (an institution has the right to rule) and a sociological side (an institution is perceived to have a right to rule) (Buchanan and Keohane 2006). Often, legitimacy is function-specific, notably as far as private governance institutions are concerned. Private regulatory actors are regarded as legitimate if they perform the problem-solving and coordination functions that they were assigned (Weber 1968). In that sense, technical expertise is a quality on which such actors can base their legitimation strategy. However, this quality alone will be necessary but not sufficient to legitimise a private regulatory actor such as an SDO if the latter performs a public purpose and in view of the public good nature of their main output (that is, the standards they adopt). Indeed, for such institutions it matters how specifically they manage to reduce transaction costs, solve coordination problems and achieve scientific consensus.

A well-known view of legitimacy is inspired by scholars working on the European political process of integration (Majone 1998; Moravcsik 2002). Legitimacy in the standardisation process in particular can relate to both participation in the standard development process (‘input legitimacy’), the design of that process and the quality of interactions within the SDOs (‘throughput legitimacy’) as well as to the standards resulting from the process (‘output legitimacy’) (Schmidt 2013). In the case of standardisation, ESO legitimacy should not only be assessed by taking into account the perceptions and behaviour of its constituents/members; rather, ESO legitimacy shall be equally based on perceptions of their principals, in this case the EU executive but also the EU legislative, including the EU governments. The latter is warranted because ESOs participate in the legislative process through their role in the New Approach.

By spelling out essential requirements that are incorporated in EU law through the adoption of harmonised standards, ESOs also produce EU law. Thus, they should be structured in accordance with democratic principles and be perceived as legitimate systems of governance when executing these functions. Such principles include the founding principles of European standardisation that the Regulation 1025/2012 mentions, i.e. coherence, transparency, openness, consensus, voluntary application, independence from special interests and efficiency. These principles align with principles for standards development identified in the realm of international trade law, notably the TBT principles for the development of international standards (Delimatsis 2015). Theoretical work on institutional legitimacy puts into spotlight deliberative interactions among stakeholders and how these promote transparency, accountability, efficacy, inclusiveness but also openness to civil society, which allows for interest intermediation and articulation (Liebert and Hans-Jorg 2009). Academic scholarship focuses on due process in private organisations that perform a public purpose beyond the national level, in the transnational sphere (Curtin and Senden 2011; Kingsbury, Krisch, and Stewart 2005; Scott, Cafaggi, and Senden 2011).

The legitimacy of standardisation as a regulatory instrument has been a contentious issue since the launch of the European New Approach. In 2016, the debate was given a new impetus by the *James Elliott* landmark judgement of the Court of Justice of the European Union *(CJEU)(C-613/14).* In this judgement, the Court ruled that the relevant harmonised standard (HS) of which the references have been published in the EU Official Journal ‘forms part of EU law’. This ruling attempts to clarify the legal status of HSs under EU law. Crucially, however, it leaves fundamental questions open, including the exact scope of the Court's jurisdiction in relation to HSs and whether there is formal delegation of powers to the ESOs.

Another related question covering access to standards concerns the role of copyright. In the *Public.Resource.Org, Inc. and Right to Know CLG v Commission (T-185/19),* the General Court of the EU recently dismissed a claim that HSs are not protected by copyright. However, in her Opinion published in June 2023, AG Medina suggested that the General Court erred in law and instead argued that HSs should not benefit from copyright protection. In finding so, AG Medina drew, among other cases, from another recent CJEU decision relating to international standards referenced in an EU legislative instrument: Based on the principle of legal certainty, the CJEU found in *Stichting Rookpreventie Jeugd and Others (C-160/20)* that in case of incorporation by reference of international standards developed by the International Organization for Standardization (ISO) in a legislative act of the EU, the standards are binding on the public generally only if these have been published in the EU Official Journal. Still, the Court avoided answering the question of whether access also includes free access to mandatory standards.

In March 2024, the Grand Chamber of CJEU got the opportunity to address this question in relation to access to four harmonised standards produced by CEN (*C-588/21 P).* Following the findings made in previous cases, including *James Elliott* and *Stichting,* the Court confirmed that HSs form part of EU law and therefore the principles of the rule of law and transparency impose in principle free access to such standards that allow for the verification of whether a given product or service complies with those standards, meeting the requirements of the EU legislation that incorporates those standards. Thus, in deviating from the General Court’s view, the Court found that basic EU principles rather support the conclusion that ensuring the disclosure of such HSs constitutes an overriding public interest that trumps any commercial interest, including that relating to intellectual property protection of CEN. Therefore, while ESO’s indeed hold copyright over their standards, once incorporated in an EU legal instrument, basic tenets of EU law require that such standards become accessible to all legal and natural persons of the EU, allowing them to ascertain their rights and obligations in order to comply with the requirements of the EU legislation. As the business model of ESOs (and by implication other SDOs which EU legislation may refer to, such as ISO standards) is challenged, heated debates on the repercussions of this case law are bound to arise.

Thus, as one can infer, the legitimacy discourse surrounding ESOs is bound to intensify for various reasons. First, because the rule of law and the principle of transparency will grow in prominence in the EU constitutional order (Senden 2020). Second, and more importantly, the scope of standardisation activities is increasingly expanding beyond traditional engineering fields to encompass ‘softer’ topics and societal concerns that cannot be exclusively covered by engineering professionals, including social responsibility, ethics, data protection and the like. Additionally, fundamental rights issues resulting from the ‘data revolution’ have put additional pressure on regulators and the standardisation community to adequately respond to and address legitimacy – and diversity-related challenges. This includes integrating new skills; ensuring diversity of voices, including gender-related questions; attracting more diverse expertise; and appealing to younger generations of experts in the standardisation process.

Exemplifying this crucial paradigm shift, the new EU Standardisation Strategy underlines the importance of taking more seriously the founding principles of the European standardisation ecosystem. More specifically, this Strategy of the European Commission calls for the modernisation of governance and the strengthening of the integrity of the European Standardisation System based on administrative and good governance principles. The European Commission highlights the need to safeguard European interests and improve accessibility and inclusiveness for small and medium enterprises, members of civil society and other users. Crucially, the Commission calls on the ESOs to consider free access to standards and other deliverables. Even so, however, it is worth noting that free access refers to output legitimacy but does little (at best) to improve input or throughput legitimacy, as described above. More concerted efforts will be needed to ensure a fair, open, and transparent standard-setting process.

### 3.1. Organisational crises as opportunities for demonstrating and enhancing resilience

ESOs are connected to the EU regulatory machinery through the New Approach, and thus cannot operate in silos. The current (geo-)political environment puts significant pressure on ESOs and constitutes a key source for internal intensive debates and potential conflicts, typically focusing on the need and the adequacy of responsive action and (non-)incremental change. In short, this episode may give rise to an organisational crisis. Such triggering events that challenge the institutional status quo; question the legitimacy, practices and business ethos of a given organisation; and call for urgent introspection, action, review and potentially radical institutional reform challenge the resilience of ESOs (Delimatsis 2023).

Conceptually, a crisis episode aims to capture low-probability yet high-impact events that threaten the fundamentals and often the very existence of an organisation and are accompanied by a common belief that decisions must be taken rapidly (Pearson and Clair 1998). An organisational crisis is an erratic event calling for swift crisis management under uncertainty to allow for recovery; otherwise they can lead to the collapse and potential extinction of an organisation.

Often, crises are not caused by a regulatory disaster, for instance, a food scandal or technical glitch leading to mass damage and complaints. Rather, crises grow gradually from an increasing sense of delegitimisation within an organisation, triggered by internal mismanagement and/or external pressure that is not seriously taken into account. Thus, a crisis incubation period that is not seriously taken up due to mistaken assumptions, information asymmetries, faulty organisational culture or unjustified optimism can develop into a full-fledged crisis that tests the crisis management reflexes of an organisation and instigates change. More often than not, as institutions such as SDOs are part of a broader organisational ecosystem where different stakeholders of private, public and hybrid nature interact, such a change will be brought about by a blend of internal troubles and exogenous forces that bring a subject of reduced resilience into a tipping point.

As institutions grow in salience (think, for instance, how ESOs grow in prominence since the launch of the New Approach and the Single Market Programme), their authority also increases, leading to contestation. In such a setting, legitimacy gaps are more explicitly articulated through discursive and behavioural practices. Such adversity calls into question an institution’s resilience. Resilience is a heavy-laden, thoroughly debated concept (Delimatsis 2023). For our purposes, we consider resilience as a dynamic concept that relates to an organisation’s pre-adversity capabilities, in-crisis capabilities and adjustment potential, and post-crisis resilient responses (Williams et al. 2017). In that sense, organisations of ever-increasing public exposure such as the ESOs will need to have dynamic and adaptive resilience strategies in place that cater for institutional adversity.

### 3.2. ‘Crises’ in ETSI

The recent changes to the legal and policy standardisation landscape in the EU provide a remarkable example of how private governance institutions respond to potential crises manifestations. The legitimacy demands placed on ETSI by the European Commission challenged ETSI’s modus operandi, requiring a substantial revision of its procedural framework. At the same time, ETSI had to adjust to the emerging regulations, such as the recently adopted AI Act,^10^ and the much-criticised SEP Regulation.^11^ These regulatory initiatives are the result of longstanding legitimacy concerns that go beyond ETSI and pertain to the EU standardisation system; yet, precisely for its unique institutional features, its openness, also to non-European influence, and its operational sector, ETSI, out of the three ESOs, is the most impacted by the imminent regulatory changes. Not less interesting, however, are ETSI’s responses to the challenges, as well as the resilience strategies it may pursue. All this makes ETSI a fitting case study to test theories relating to organisational crises and resilience (Delimatsis and Verghese 2024). To that end, the following sections introduce the legal and policy instruments that served as catalysts of the ‘crises’ situations and required reactions from ETSI, and set the context for analysing ETSI’s legitimacy and resilience.

#### 3.2.1. The 2022 standardisation strategy

The rapid evolution of standards as strategic and political instruments presents new challenges that require adequate responses. To address these challenges, the European Commission adopted a New Standardisation Strategy in February 2022. Together with the Strategy, the Commission introduced a proposal with amendments to the Regulation on European standardisation^12^ and a report on the implementation of the EU Standardisation Regulation.^13^ The 2022 Standardisation Strategy identified ‘standardisation urgencies ’ – areas that need standards to lessen dependencies on foreign actors but also where the EU can manifest its global leadership, which included chips certification in terms of security, hydrogen value chain and digital technologies. Acknowledging that standards do not only deal with technical components, but also project the EU democratic values and interests at the global level, the Commission proposed a number of action points to improve the integrity and inclusiveness of the European standardisation system.

In particular, the Commission was concerned about the potential influence of foreign companies on European standardisation. Indeed, the growing presence of Chinese firms in the global standardisation arena has not gone unnoticed (Baron and Kanevskaia 2021; Cantero Gamito 2021; Nanni 2021). This development is also echoed in ETSI, where Huawei, the Chinese champion in the technology sector, was a founding member of ETSI’s Industry Specification Group on Securing AI and initiating the (unsuccessful) attempt to develop centralised Internet protocols (Perarnaud and Rossi 2024). The EU is especially uncomfortable with the growing Chinese footprint on European standards due to the sharp difference between the EU’s and China’s norms for fundamental rights protection, as well as the long-standing link of leading Chinese companies with the Chinese Communist Party (Rühlig 2022). As an ESO with a global reach that allows foreign companies not only to become ETSI members, but also to vote on important standardisation decisions and be in charge of ETSI’s management,^14^ ETSI got the large share of the blame for letting European standards to be unduly influenced by foreign interests.

To mitigate these concerns, the New Standardisation Strategy proposed to strengthen the role of National Standards Bodies (NSBs), that is, the standardisation committees of the EU and EEA Member States. The amendments introduced by the Regulation 2022/2480 requested the ESOs to ensure that only NSBs can take decisions concerning harmonised standards, including those on the acceptance of standardisation requests or new work items, and on the adoption, revision or withdrawal of standards.^15^ These amendments were proposed for the three ESOs, but it isETSI’s voting rights that had to be adjusted the most so that (foreign) companies can no longer have decision-making power on standards that support European policy (EC 2022a).

Needless to say, the requested changes have been contested by companies outside the EU and EEA Member States, but also by some European actors. For instance, the European Association for the Co-ordination of Consumer Representation in Standardisation (ANEC) pointed out that the positions taken by NSBs also risk being influenced by companies that are based outside the EU and EEA but are active in their Member States’ national standardisation processes. Accordingly, the ETSI reform is meaningless without a mirror obligation for NSBs to ensure that their decision-making is also driven by European interests (ANEC 2022). Remarkably, the emphasis on the NSBs also entails that the NSBs, and not the ESOs, as per the Commission’s 2003 Cooperation Guidelines, now carry responsibility for interest representation in European standardisation. This raises the question whether ESOs’ other obligations, including stakeholder participation,^16^ are rendered meaningless due to the strong reliance on NSBs.

The requested reform is an illustrative example of a crisis moment for ETSI. Not only does it reveal the Commission’s doubts of ETSI’s ‘European’ character and its commitment to European values, but it also highlights the Commission’s distrust in the ETSI governance system that thrived on its openness and orientation towards global markets (see, in this regard, Temple 2024). Whereas the proposed amendments were introduced for all three ESOs, ETSI is arguably the most affected by the required changes, which challenge its well-established working methods. Being globally oriented and allowing direct participation of industry interests, as opposed to their representation through NSBs, ETSI arguably understands inclusiveness differently from other ESOs or, even the European Commission itself. In effect, ETSI’s model of openness and inclusiveness has proved to clash with the objective to pursue European values.

Despite the aforementioned, the reform was accepted by all three ESOs, leading to the critical amendments to their governance frameworks in June/July 2023. CEN/CENELEC internal regulations introduced the system of ‘double counting of votes,’ where votes of the NSBs and all voting members will be counted and displayed separately; NSBs are to take final decisions in case discrepancies occur, but only after having addressed those with other voting members.^17^ Also ETSI limited the decision-making on HSs to the NSBs,^18^ arguably shifting power dynamics between ETSI membership. Since the actual technical work on standards is still carried out predominantly by the industry members, there is a growing disconnect between members accepting the Commission’s standardisation request and those involved in standardisation processes. Curiously, the version of the ETSI Statutes of December 2023 replaced its scope of activities from ‘harmonised standards’ with ‘globally accepted standards’.^19^ It may be speculated that while ETSI is not yet ready to lose its ESO status, this seemingly minor change in wording is an indication of ETSI’s intent to assert itself as a global SDO in the future.

#### 3.2.2. New and proposed EU legislation

Technological developments of the last decades are commonly known to have transformed many sectors. This also had a consequence for standardisation. Firstly, innovations such as 5G and the Internet of Things (IoT) led to the situation where telecommunications and Internet standardisation, while rooted in different cultural and operational traditions, are effectively merging (ten Oever 2023). Generally charged with political implications (DeNardis 2009), both sectors are of strategic importance to the EU, and therefore likely to be subjected to the additional scrutiny from the European Commission in the years to come.

Secondly, the success of IoT technologies brought new actors to the telecom standardisation game, including the rapidly growing automotive sector (Heiden, Padilla, and Peters 2021). In many ways, the involvement of new players challenges the current standardisaton practices, calling for the re-assessment of the existing legislative framework.

Thirdly, the increasing use of (personal) data and algorithms spurred the emergence of new regulatory fields as well as the need to revisit the regulation of the existing ones. In this regard, the reform of the European standardisation system cannot be seen in isolation from the EU’s recent efforts to regulate digital markets and to ensure its digital sovereignty, on the one hand, and to establish its normative power globally (often discussed in the context of the ‘Brussels effect’ (Bradford 2020; Perarnaud and Rossi 2024)), on the other. It is in part for these reasons that the EU has recently adopted a new generation of ‘international hybrid digital policies’ (Broeders, Cristiano, and Kaminska 2023) that aim to safeguard the digital single market while also protecting the fundamental rights of EU citizens. By promoting its values through economic policies, the EU aims not only to preserve its regulatory and normative power, but also to become a geostrategic actor (Broeders, Cristiano, and Kaminska 2023) and create fertile ground for responsible and trustworthy innovation.

Fourthly, the new generation of EU legislation draws heavily on the well-established harmonisation techniques, and in particular, the New Approach. Somewhat ironically, the EU regulatory efforts grant even more power to private actors, including the ESOs and conformity assessment bodies. Indeed, due to the regulatory complexity and the need of specialised knowledge, State-driven regulation often proves insufficient for the emerging regulatory fields (Büthe and Mattli 2011). (Harmonised) standards as tools that demonstrate compliance with EU legislation are featured in a number of recently adopted EU Regulations, including the Digital Markets Act,^20^ the Digital Services Act,^21^ which refers to voluntary standards of both European and international standards organisations, and the proposed Data Act,^22^ in relation to HSs for data spaces and for interoperability of data processing services.

Perhaps the most intriguing example is the inclusion of HSs in the AI Act. As explained by Cantero Gamito in this Special Issue (Cantero Gamito 2024), the use of the New Approach technique presents shortcomings when it comes to securing fundamental rights. The text of the provision on the harmonised standards has been adjusted several times in the recent rounds of negotiations. Among others, the current version requires the Commission to take on board the opinions of the AI Advisory Forum and the relevant stakeholders when issuing a request to produce HSs introduces a reporting obligation for the ESOs with regard to their fulfilment of the objectives set out in this provision, and requires actors involved in standardisation processes to take into account existent international standards that are ‘consistent with the EU’s values, fundamental rights and interests’.^23^ Still, the question remains whether the New Approach was an adequate regulatory choice in the context of fundamental rights (Almada and Petit 2023). This is particularly so since, being a pioneering attempt to regulate AI, the requirements of the AI Act may be uptaken by other jurisdictions, notably the United States (US), transposing the rather limited protection of fundamental rights beyond the EU (Almada and Radu 2024).

For ETSI, it may not be the imminent adoption of the AI itself that created a situation akin to a ‘crisis’, but the first standardisation request filed by the Commission under the AI Act. In particular, the Commission directed its request to develop HSs to CEN and CENELEC, leaving ETSI with a merely advisory role.^24^ This ‘exclusion’ is allegedly rooted in the longstanding critique on the ETSI governance model that allowed foreign companies to partake in critical decisions on harmonised standards.^25^ Recently, some sources have suggested the possibility of ETSI to be also excluded from the request to develop HSs for the Cyber Resilience Act (CRA),^26^ although this information has not been confirmed as of yet. What is at stake is not only ETSI’s status as an ESO, but also its market reputation: should ETSI deviate from its industry-oriented, open participation model, praised by many market stakeholders to secure its involvement in defining European AI policy? While the answer to this question seems obvious due to the recent procedural changes to the ETSI Directives, it may be less so in the future if ETSI’s exclusion from the development of HSs becomes a recurring practice.

Furthermore, the AI Act provides a possibility for the Commission to establish ‘common specifications’ should an ESO reject its standardisation request or produce an unsatisfactory standard.^27^ While the ESOs always have a right to deny a request for a harmonised standard, the threat of common specifications may result in a sort of a carrot and stick approach where the Commission ensures that HSs indeed respond to the European policy needs. This also limits the possibility of ESOs’ strategically denying standardisation requests.

Another potential ‘crisis’ has recently been caused by the proposed change of the EU regulatory landscape on Standard Essential Patents (SEPs). As such, SEPs are one of the most salient topics of technology standardisation (Kanevskaia 2023). Owning a patent that is essential for standards’ functionality and correct implementation is a source of power and potential abuse, but it is also the driving force behind innovation. Typically, SEP owners agree to license their technology on Fair, Reasonable and Non-Discriminatory (FRAND) terms, leaving the exact definitions of royalty rates to the licensing parties. This SEP licensing practice has been both praised for providing contractual freedom as well as criticised for the lack of transparency and legal certainty for SEP implementers, especially small and medium enterprises (SMEs). Attempts to provide a definition of FRAND have been successful in some SDOs, notably the Institute of Electrical and Electronics Engineers (IEEE), but did not make it in ETSI.

As a response to the pertinent critique of the EU licensing landscape, the European Commission presented a proposal for a SEP Regulation in April 2023. The proposal requires (potential) SEP owners to register their patents in a designated SEP register with the EU Intellectual Property Office (EUIPO), which will then conduct essential checks, set up an aggregate royalty rate and make individual FRAND determination – processes that have been traditionally carried out through market-based negotiations. It will also require licensing parties to initiate a non-binding conciliation procedure with the EUIPO prior to initiating national litigation.

The Commission’s proposal was followed by an unparalleled flood of critique, with companies submitting counter proposals^28^ and lobbying heavily to get the proposed Regulation outvoted by the European Parliament.^29^ The proposed Regulation has been heavily criticised for, among others, its alleged non-proportionality, lack of balance and unjustified regulatory intervention; many commentators have also suggested that if adopted, SEP Regulation will deter innovative firms from the EU, undermining European global competitiveness (Baron 2023; Galli, Nikolic, and Botta 2023).

For ETSI, the proposed Regulation creates a division between members who are in its favour, mostly patent implementers, and those who are against, making it nearly impossible for the institution to speak with one voice. Indeed, to this day, ETSI has issued no position letter or statement regarding the proposed SEP Regulation. This is even more distressing because most licensing disputes are bound to arise in ETSI rather than CEN or CENELEC due to the ESOs’ operational sectors. Furthermore, the proposed SEP Regulation also calls into question the established practices around SEP disclosure and licensing: ETSI maintains its own Intellectual Property (IPR) database for patents that have been declared as (potentially) essentially to its standards, and ETSI’s IPR Policy does not provide a definition of FRAND.

## 4. Contributions summaries

To give due account to the multifariousness of legitimacy and resilience, this Special Issue presents a collection of multidisciplinary contributions that look at ETSI’s legitimacy and resilience through different lenses. The contributions are organised around four themes and are followed by a commentary, reflecting on the papers, adding new perspectives, and suggesting further paths for research.

The first three papers are centred around ETSI’s historical evolution and explanation of its organisational resilience. Michelle Egan opens the discussion by providing a historical account of ETSI’s creation and evolution in a (transatlantic) context. Egan’s contribution contrasts the Commission’s endeavour behind ETSI’s creation in 1988, namely the need to speed up and strengthen EU standardisation in the domain of telecommunications, with the Commission’s current normative agenda that aims for standards to reflect European and democratic values. Highlighting the importance of transatlantic cooperation in standardisation, the author draws the readers’ attention to the US Standardisation Strategy adopted in 2023. While bearing some similarities with the EU strategic position, notably on international leadership in standards and concern with growing Chinese influence in technology standardisation, the US strategy is different from the EU in its geoeconomic, outward oriented approach. Egan argues that the EU’s focus on internal governance and its shift from the strong private, industry-led standards processes is at odds with ETSI’s globally oriented model of standard setting and therefore, risks undermining ETSI’s leadership and legitimacy as a developer of technology standards.

Delimatsis and Verghese add to this historical perspective by addressing ETSI’s evolution as a result of its dynamic interactions with the EC over the course of 30 years of ETSI’s existence. The authors provide an analytical framework that focuses on such interactions during crisis moments in the past, when ETSI faced challenges and (internal and external) legitimacy pressures, and its resilience was questioned. They identify three resilience strategies enacted by ETSI vis a vis the EU Commission that helped it overcome these critical moments, namely: contestation, orchestration and collaboration. The authors provide empirical accounts of three key episodes in ETSI’s history when these strategies surfaced and find that these strategies were not only resilience-enhancing but also legitimacy-inducing, reinforcing further ETSI’s position as the preferred ICT standard setter at the EU level but also as a reliable global partner in the ICT landscape.

The commentary by Loyau and Bijlmakers addresses the contributions by Egan and Delimatsis and Verghese, bringing practical insights into the academic discussions. The authors focus on ETSI’s unique organisational features and identify three key traits that contributed to ETSI’s organisational resilience: its institutional set-up, the quality of its standards, and the ETSI IPR policy. Drawing on historical examples, the authors demonstrate how these traits were activated and leveraged during critical moments, and insulated ETSI from pressures and (legitimacy) demands, including also those from the EC.

The following series of papers unpacks the legitimacy of ETSI and its governance in particular, conceptualising and engaging with legitimacy theories from law and political science.

De Vries opens the discussion by providing a historical perspective to clarify the EU’s current questioning of ETSI’s legitimacy, and the importance of European values. De Vries notes how values played an important role in the Commission’s striving for European unification, and how European standardisation in contributing to this objective was driven by political and cultural imperatives. The author, after having shed light on the concept of legitimacy and its multifacetedness, proposes that a solution to the EU’s legitimacy concerns not be sought not in participation (input or throughput legitimacy) but in the use of standards (output legitimacy). According to De Vries, this solution would involve public regulators applying value-based criteria in their selection of standards for use, while ETSI would not have to undergo any changes Standard developers in ETSI may want to take these criteria into account, as doing so would increase the use and legitimacy of their standards, and give them a competitive edge.

Volpato and Eliantonio further contribute to the debate on ETSI’s legitimacy, examining the involvement of societal stakeholders within ETSI’s standard-setting processes, through the lens of throughput legitimacy. More specifically, the authors examine ETSI’s membership and voting rules to discuss the possibilities of the so-called Annex III organisations to effectively participate. The authors find that whilst having full membership status and corresponding formal rights to participate, they face practical limitations related to a lack of resources and expertise. ETSI’s voting rules and its definition of consensus create further obstacles, giving chairs discretion to determine when a ‘sustained opposition’ exists, consensus has been reached and a vote is necessary. The authors identify various issues that undermine ETSI’s institutional and constructive throughput legitimacy. Recent reforms have introduced mechanisms that partially addressed these concerns; it remains to be seen whether these responses will improve the effectiveness of Annex III organisation’s participation in ETSI.

Temple shares a view on ETSI’s legitimacy that was held by its founders. According to the author, the legitimacy of ETSI standards should be understood in relation to both its openness and the acceptance of its standards by the market. Openness has different meanings; the right for anyone who can afford a reasonable membership fee to participate in standard development, the smallest company having the same right as the largest global company to be heard, and the right of anyone to implement the standard. The author argues that ETSI’s industrial ecosystem in its entirety being represented in its standardisation process has contributed to its success. Temple defines success as such procedures having created the best conditions for standards to arise out of a so-called ‘doom loop’. According to Temple, the market should be the ultimate arbiter of ETSI’s legitimacy.

Bijlmakers closes this part of the Special Issue by reflecting in her commentary on the three contributions mentioned above. According to Bijlmakers, these contributions are illustrative of the multifacetedness of legitimacy in standardization, assessing ETSI’s performance based on different normative legitimacy standards and along different legitimacy objectives/ideals. This contribution adds a dynamic account of legitimacy to unpack current dynamics within ETSI’s decision-making. Questions for further research are raised about ETSI’s autonomy to make decisions free from (legal) restraints, and how this episode may offer opportunities for ETSI to increase its legitimacy and gain rulemaking influence as an ESO in the public arena, and globally.

The two following papers provide an empirical account of organisational resilience.

The article by Stanojević examines empirically the organisational resilience strategies used by ETSI during significant moments in its history when the organisation experienced a threat to its functioning and/or performance. A taxonomy of resilience strategies is provided, which differentiates strategies along two different dimensions, whether they were adopted in anticipation of or reaction to such a disturbance, and the locus of the strategic effort, be it internal or external to the organisation. The author reflects on how ETSI’s use of these strategies contributed to the organisation’s continued thriving over time amidst changing conditions, attesting to its resilience. Standardisation politics can help explain ETSI’s organisational responses and outcomes. For instance, whilst ETSI identified the Commission’s criticism of industry’s (dominant) influence in the organisation as a disturbance and used several resilience strategies to address them (e.g. discussion, leadership and cooperation with public actors), criticism from academia and citizen groups about human health concerns were dismissed. ETSI’s use of resilience strategies was not necessarily beneficial for its relations with socio-ecological systems. At least in some cases, strategies used addressed external criticism about participation within ETSI of underrepresented stakeholders superficially (pro forma). ETSI’s continued success despite such strategies not addressing issues at its core opens new avenues for future research.

Bekkers and Lazaj examine empirically ETSI’s voting and its significance as a decision-making mechanism versus consensus. Voting has occupied a central place in current discussions about ETSI’s governance. As written in its procedures, ETSI strives to reach decisions by consensus but can take decisions by way of voting, relying on one of its three voting procedures: Weighted National Voting, Full Member Individual Voting or Weighted Individual Voting. The authors analyse decision making by ETSI’s General Assembly, between 1988 until 2022, to determine how often, how and when voting was used. The authors find that only 4.6% of the decisions were taken by voting, and that most decisions were not contentious nor of great significance from a governance perspective. The authors argue that voting within ETSI may have received more attention than it deserves, and that a better understanding of how actual decision-making by consensus works may be of higher importance, allowing a more realistic assessment of ETSI achievements and pitfalls regarding its decision-making processes.

Wiegmann comments on Stanojević and Bekkers and Lazaj’s contributions. According to the author, the European Commission may have overestimated the implications of the EU Standardisation Strategy for ETSI. From Bekkers and Lazaj’s finding that only a small share of all decisions about ETSI’s governance is reached through voting, the author notes that ETSI may be closer to the European Commission’s ideal of ‘integrity, inclusiveness, and accessibility’ than presumed. The author questions whether the ‘tied and tested’ resilience strategies identified by Stanojević will be effective in making the European Commission aware of this. Wiegmann views relevance for both works beyond ETSI, to inform broader discourse on standardisation, and raising questions for future research about how Network Administrative Organizations (NAOs) respond and adapt to challenges from within and outside their network.

The last series of papers is about how legitimacy and resilience of ETSI and in the broader sense, of the EU standardisation, is to be tested by the future regulations of emerging technologies.

Cantero Gamito examines the shortcomings of the New Approach for the AI Act and ETSI’s role in AI standardisation. The proposed EU AI Act provides a legal framework for ensuring AI systems are trustworthy and respect fundamental rights, safety and ethical principles. The EU AI act relies on harmonised standards to specify essential requirements for the design and development of high-risk AI systems. Manufacturers may rely on these harmonised standards to demonstrate compliance with these requirements, and obtain a declaration of conformity and a CE marking. The task of developing these standards is delegated to ESOs, which de facto entails translating normative choices into technical requirements that reflect EU values. The standards will have significant influence in shaping the AI landscape in the EU and perhaps beyond. This has revived old discussions about the legitimacy of delegation of rulemaking concerning fundamental rights, and the democratic accountability of EU standardisation. ETSI’s exclusion from the latest draft standardisation request signals that ESOs must have inclusive standard setting processes in place that effectively balance technical considerations with societal and ethical norms. The author reflects on the implications of this decision for AI governance and the broader digital sphere.

Kamara examines standardisation and law in the domain of cyber security resilience. ETSI and the other ESOs develop cyber security technical standards in parallel. Who develops these standards and with which processes matter for their legitimacy, according to the author. The European Commission is setting out cyber security legislations and policies to improve responsiveness to cyber security incidents in the EU (EU cyber resilience). An important development is the forthcoming CRA, a horizontal EU cybersecurity product law that aims to ensure that products with digital elements that are placed on the market are cyber secure. European standards are needed to operationalise essential requirements in the CRA, relating to these products and evaluation methods for risks and assurance levels. As a further expansion of cyber-security standardisation activities can be anticipated, there are risks of duplication of efforts and incoherence among European standards developed by the ESOs. Whilst there is a need for cooperation by ETSI with CEN and CENELEC to overcome these risks, formal channels appear to be underused. Kamara argues that a mode of co-creation of HSs with broad representation of interests would best respond to the needs of cyber resilience in the EU.

The commentary by Kanevskaia underscores the importance of power dynamics . The author argues that rather than increasing the legitimacy of private rulemaking, the EU Standardisation Strategy was an attempt by the Commission to regain its power and control over private regulatory actors. The expansion of standardisation activities has resulted in a duplication of efforts and institutional competition between the ESO, with implications for the legitimacy of the European standardisation system. According to the author, the EU, by excluding it from the AI standardisation request, ‘demoted’ ETSI as an ESO, because it was not satisfied with its governance. Whilst this may be welcomed from a public law perspective, such exertion of power by the EU Commission is undesirable and possibly detrimental to the EU’s position in standardising the technology sector.

## 5. Challenges for ETSI’s legitimacy and resilience

The European policy on digitalisation and innovation is moving at a fast pace. ESOs, and ETSI in particular, are bound to play a key role in the implementation of the EU digital decade initiative and the EU industrial strategy. The new regulatory landscape within the EU, but also the volatile geopolitical environment will test ETSI’s resilience not only as an ESO, but also as a global SDO, requiring ETSI to adapt its traditional way of working. These two different roles hinge upon two different types of legitimacy. As an ESO, ETSI is tightly linked to EU law and policy-making, with the European Commission being the main arbiter of its legitimacy. As a global SDO, ETSI is an established actor in the field of technology whose legitimacy manifests through including, rather than excluding, a wide range of companies. It is not that these two types of legitimacy cannot co-exist; on the contrary, an ESO supported by the European Commission makes a strong case to become a powerful global actor. To stay resilient, ETSI needs to find a way to navigate through these – often competing – legitimacy demands. But ETSI will not be able to do it alone: meaningful support from its market participants but also the European Commission will be essential in this delicate mission.

## Notes

1 Council Resolution of 7 May 1985 on a New Approach to Technical Harmonisation and Standards, OJ C 136/1, 7 May 1985.

2 Decision No 768/2008/EC of the European Parliament and of the Council of 9 July 2008 on a common framework for the marketing of products, and repealing Council Decision 93/465/EEC.

3 General Guidelines for the Cooperation between CEN, Cenelec and ETSI and the European Commission and the European Free Trade Association, OJ nC 91/04, 28 March 2003.

4 See also Regulation (EU) No 1025/2012 of the European Parliament and of the Council on European standardisation, OJ L 316/12, 25 October 2012 (‘The EU Standardization Regulation’).

5 General Guidelines for the Cooperation between CEN, Cenelec and ETSI and the European Commission and the European Free Trade Association, OJ nC 91/04, 28 March 2003.

6 https://www.cencenelec.eu/areas-of-work/cen-sectors/.

7 https://www.cencenelec.eu/areas-of-work/cenelec-sectors/.

8 https://www.cencenelec.eu/areas-of-work/cen-cenelec-topics/smart-grids-and-meters/cen-cenelec-etsi-coordination-group-on-smart-grids-cg-sg/.

9 E.g. MoU with Euro-Asian Council for Standardization, Metrology and Certification (EASC) (24 May 2012) available at https://easc.by/images/document/sotrudnichestvo/cce-memorandum.pdf.

10 European Commission, Proposal for a Regulation of the European Parliament and of the Council laying down harmonised rules on artificial intelligence (‘AI Act’), COM (2021) 206 final.

11 European Commission, Proposal for a regulation of the European Parliament and of the Council on standard essential patents and amending Regulation (EU) 2017/1001, COM (2023) 232 (27 April 2023).

12 European Commission, ‘Proposal for a Regulation of the European Parliament and of the Council amending Regulation (EU) No 1025/2012 as regards the decisions of European standardisation organisations concerning European standards and European standardization deliverables’ COM (2022) 32 final. Approved by the European Parliament in October 2022.

13 European Commission, ‘Report from the Commission to the European Parliament and Council on the implementation of the Regulation (EU) No 1025/2012 from 2016 to 2020’ COM (2022) 30 final.

14 The current ETSI board is chaired by a representative of a US firm and counts many US and UK companies. https://portal.etsi.org/TB-SiteMap/BOARD/Board-Members.

15 Regulation 2022/2480 of the European Parliament and of the Council, amending Regulation (EU) No 1025/2012 as regards decisions of European standardisation organisations concerning European standards and European standardisation deliverables, 14 December 2022, OJ L 323/1.

16 Regulation 1025/2012, Article 5.

17 CEN/CENELEC Internal Regulations, Part 2. Common Rules for Standardization Work (July 2023), Clause 6.4.

18 ETSI Directives, V 48 (December 2023), Article 13.

19 ‘The activities of the Institute shall contribute to the production and the promotion of new globally accepted world-wide standards and furthermore shall build upon world-wide standards, existing or in preparation’ ETSI Directives, V48.

20 Regulation 2022/1925 of the European Parliament and of the Council on contestable and fair markets in the digital sector, 14 September 2022, OJ L 265/1, Article 48.

21 Regulation 2022/2065 of the European Parliament and of the Council on a Single Market For Digital Services, 19 October 2022, OJ L 277/1, Article 44.

22 European Commission, Proposal for a Regulation of the European Parliament and of the Council on harmonised rules on fair access to and use of data, 23 February 2022, COM/2022/68 final, Articles 28 and 29.

23 Art 40 (1c) of version 2021/0106 (COD) leaked in January 2024. The latter requirement was most probably added to echo the requirement of the WTO TBT Agreement that national technical regulations should be based on international standards (Article 2.4 TBT), in anticipation of the emerging AI standards developed by the International Organization for Standardization and the International Telecommunications Union.

24 European Commission, Draft standardisation request to the European Standardisation Organisations in support of safe and trustworthy artificial intelligence. European Commission, 5 December 2022, available at https://ec.europa.eu/docsroom/documents/52376.

25 Luca Bertuzzi, Commission leaves European standardisation body out of AI standard-setting, EURACTIV (9 December 2022), available at https://www.euractiv.com/section/artificial-intelligence/news/commission-leaves-european-standardisation-body-out-of-ai-standard-setting/.

26 Luca Bertuzzi, European standardisation organisation’s leadership election embarrasses EU Commission, EURACTIV (20 November 2023), available at https://www.euractiv.com/section/industrial-strategy/news/european-standardisation-organisations-leadership-election-embarrasses-eu-commission/.

27 AI Act, Article 41.

28 See, among others, Kerry Miller, ‘Commission’s patents proposal doesn’t have to be so damaging’ Politico, 12 December 2023, available at https://www.politico.eu/sponsored-content/commissions-patents-proposal-doesnt-have-to-be-so-damaging/.

29 Maura O’Malley, ‘Group Including Nokia and InterDigital urges MEPs to vote against EU SEP reforms’, The Global Legal Post, 27 February 2024, https://www.globallegalpost.com/news/group-including-nokia-and-interdigital-urges-meps-to-vote-against-eu-sep-reforms-2101284277.
